# Chitinase Dependent Control of Protozoan Cyst Burden in the Brain

**DOI:** 10.1371/journal.ppat.1002990

**Published:** 2012-11-29

**Authors:** J. Philip Nance, Kevin M. Vannella, Danielle Worth, Clément David, David Carter, Shahani Noor, Cedric Hubeau, Lori Fitz, Thomas E. Lane, Thomas A. Wynn, Emma H. Wilson

**Affiliations:** 1 Division of Biomedical Sciences, University of California, Riverside, California, United States of America; 2 National Institute of Allergy and Infectious Diseases, National Institutes of Health, Bethesda, Maryland, United States of America; 3 Institute for Integrative Genome Biology, University of California, Riverside, California, United States of America; 4 Department of Inflammation and Immunology, Pfizer, Cambridge, Massachusetts, United States of America; 5 Department of Molecular Biology and Biochemistry, Institute for Immunology, University of California, Irvine, Irvine, California, United States of America; Cornell University, United States of America

## Abstract

Chronic infections represent a continuous battle between the host's immune system and pathogen replication. Many protozoan parasites have evolved a cyst lifecycle stage that provides it with increased protection from environmental degradation as well as endogenous host mechanisms of attack. In the case of *Toxoplasma gondii*, these cysts are predominantly found in the immune protected brain making clearance of the parasite more difficult and resulting in a lifelong infection. Currently, little is known about the nature of the immune response stimulated by the presence of these cysts or how they are able to propagate. Here we establish a novel chitinase-dependent mechanism of cyst control in the infected brain. Despite a dominant Th1 immune response during Toxoplasma infection there exists a population of alternatively activated macrophages (AAMØ) in the infected CNS. These cells are capable of cyst lysis via the production of AMCase as revealed by live imaging, and this chitinase is necessary for protective immunity within the CNS. These data demonstrate chitinase activity in the brain in response to a protozoan pathogen and provide a novel mechanism to facilitate cyst clearance during chronic infections.

## Author Summary

Described here is a novel mechanism of protozoan cyst clearance in the CNS during chronic infection. These data show the presence of a population of alternatively activated macrophages in the brain that secrete the active chitinase, AMCase, in response to chitin in the cyst wall. Using both chemical and genetic inhibition in vitro, it is revealed that this enzyme is required for efficient degradation and destruction of the cyst. The necessity for AMCase is demonstrated in vivo, as the absence of the enzyme resulted in a significant increase in cyst burden and decrease in survival during chronic infection. Together, these data identify an important mechanism of parasite control and cyst clearance in the CNS. Currently, no therapies exist that lead to the total clearance of this parasite from the brain. Therefore, developing an understanding of the natural mechanisms of cyst clearance has the potential to lead to new and effective therapies for this and other chronic infections.

## Introduction

The brain has unique structures in place to limit access of immune cells and molecules. Although this can provide protection against an overambitious inflammatory response it may also lead to the high prevalence of latent and chronic infections that can persist at this site. Removal of such pathogens has its own particular problems in an organ dense with sensitive neurons and stringent gateways for immune cell infiltration. *Toxoplasma gondii* is a common intracellular protozoan parasite that forms a chronic infection in the brain for the lifetime of the host. The infection is controlled, in part, through the effector mechanisms of macrophages that result in the conversion of fast replicating tachyzoites to the slow replicating, cyst forming bradyzoites [1]–[3]. Cysts can form in all tissues but exist predominantly in the brain for the lifetime of the host requiring a continuous immune response to prevent cyst reactivation and Toxoplasmic encephalitis, a common cause of AIDS related fatalities [4], [5]. The infection-induced immune response in the brain consists of activated CNS resident cells including astrocytes and microglia, infiltrating CD4+ and CD8+ T cells, peripheral macrophages and substantial tissue remodeling [6]–[8]. Such immune activity in the brain is often associated with a pathological outcome yet despite the high prevalence of infection Toxoplasma is seemingly controlled without adverse neurological damage. The mechanisms that are involved in the trafficking and control of such a potentially pathological immune response within the CNS are only beginning to be understood [6], [8]–[11]. The cyst and cyst-forming bradyzoites are poorly immunogenic [12], [13] and although we have known for some time that T cells are required to prevent cyst reactivation [4], [5], [14], very little is understood about the biology of this structure in the brain. Although anti-Toxoplasma drugs are available that efficiently control the tachyzoite, there are as yet no therapies available that can effectively remove the cyst form of the parasite. Thus, the continuous presence of Toxoplasma cysts in the brain presents a critical and constant danger for the immune compromised patient.

It is widely believed that cysts remain intracellular within neurons possibly minimizing their contact with host defense systems [15]. However it has been known for some time that cyst burden reaches a peak, declines and becomes stable over time pointing to some form of effector mechanism that can target this stage of the parasite [16]. Studies have implicated CD8+ T cell production of perforin in cyst clearance with perforin deficient mice exhibiting higher cyst burden and susceptibility at the chronic stage of infection [17], [18]. Nevertheless, histological analysis from these studies as well as recent live imaging of cell interactions in the CNS [19] demonstrates monocyte accumulation and contact with cysts.

In recent years, our understanding of macrophages has expanded and we now appreciate these cells' remarkable plasticity. Thus, although whole populations of macrophages can become polarized to classical or alternative phenotypes associated with protection against protozoan and helminth pathogens respectively, the ability to respond and adapt to local stimuli in the environment is paramount [20]–[24]. The role of classically activated macrophages in the control of *T. gondii* infection is well documented. These cells are a source of IL-12, reactive oxygen and nitrogen species, and GTPases that enable the direct killing of the parasite [6], [25]–[30]. However, here we describe a population of CXCR3+ macrophages in the brain following *T. gondii* infection. These cells express the scavenger receptors MMR and stabilin-1 and produce arginase in response to the presence of Toxoplasma cysts. In addition to these traditional signs of alternative activation, these studies demonstrate that macrophages respond to chitin present in the cyst wall and produce the true mammalian chitinase, AMCase. Finally we show that this chitinase activity destroys cysts and is essential for the control of cyst burden within the chronically infected brain.

## Results

### A population of AAMØ in the *T. gondii* infected brain

**Figure 1:**
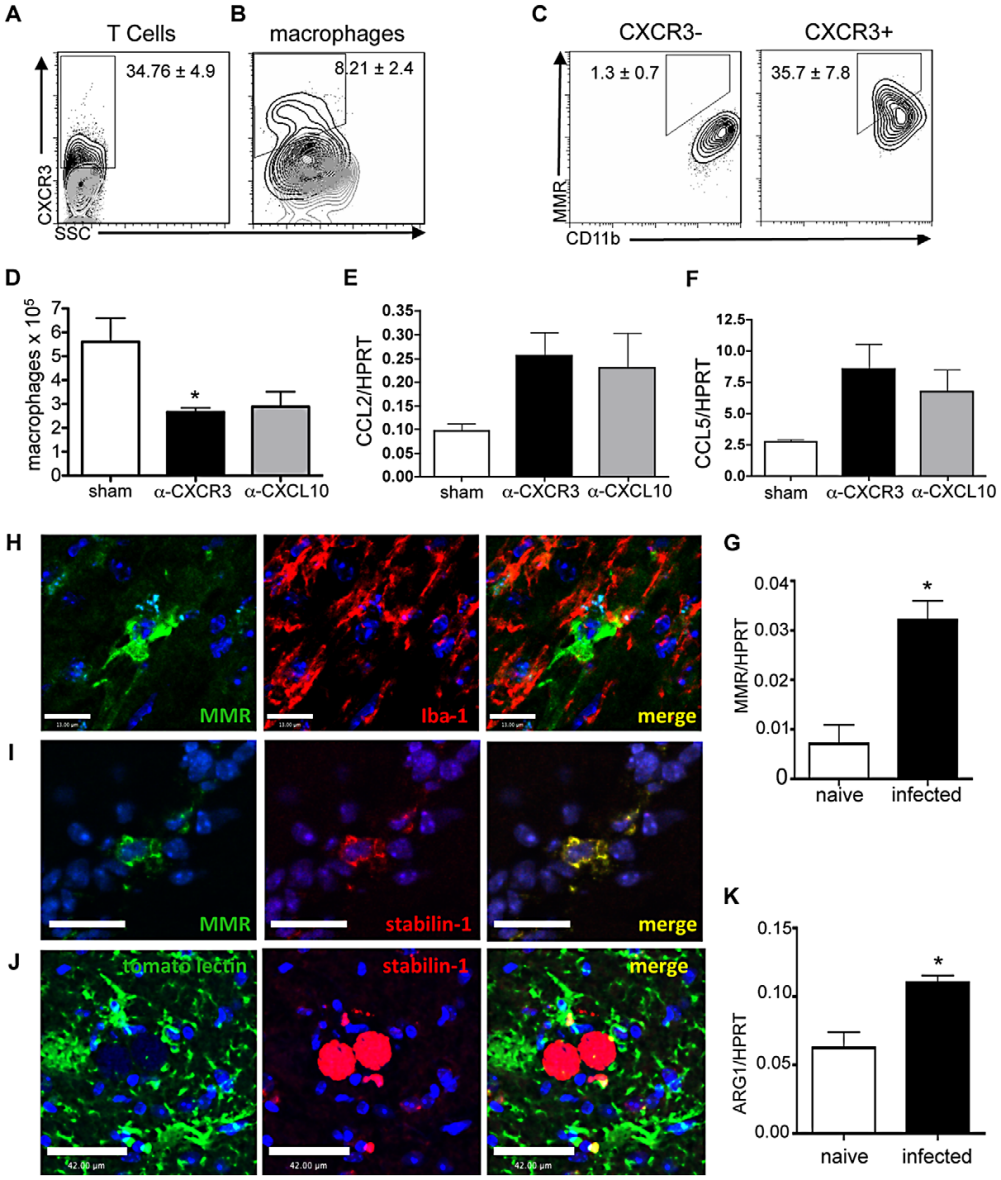
Alternatively activated macrophages in the brain during chronic *T. gondii* infection. (A–C) C57Bl/6 mice were infected with the Me49 strain of *T. gondii* and sacrificed on day 28 post-infection (p.i.). Brain mononuclear cells (BMNC) were isolated and analyzed for surface expression of CXCR3 and MMR. Mean ± SEM of percentage of CXCR3+ cells as a proportion of (A) CD3+ T cells (B) CD45^hi^/CD11b+ macrophages. (C) Mean ± SEM percentage MMR positive cells from CXCR3− (left) and CXCR3+ (right) macrophages. (D–F) C57Bl/6 mice were infected with the Me49 strain of *T. gondii*. Neutralizing antibodies for CXCR3 and CXCL10 were administered beginning at 21 days post infection and mice were sacrificed on day 28 after infection. (D) Absolute counts of macrophage populations were obtained from the proportion of CD45^hi^/CD11b+ BMNCs multiplied by the total number of live BMNCs. (E–F) RNA was isolated and reverse transcribed and cDNA was analyzed for levels of CCL2 (E) and CCL5 (F) transcript using qRT-PCR. (G) BMNCs were isolated from mice at 4 weeks p.i. and CD11b+ cells were magnetically purified by positive selection. RNA was isolated and reverse transcribed and cDNA was analyzed for levels of MMR transcript using qRT-PCR. Results are shown as absolute quantitation of MMR using a standard curve as a ratio to HPRT. (H–J) Confocal fluorescence microscopy of 20 µm brain slices taken from mice at 4 weeks post infection. (H) Panels from left to right show MMR (green), Iba-1+ macrophages (red), and merged images in the infected brain with DAPI (blue) scale bar = 13 µm. (I) Panels from left to right show MMR (green), Stabilin1 (red), and merged images in the infected brain with DAPI (blue) scale bar = 21 µm. (J) Panels from left to right show tomato lectin (green), stabilin1 (red), and merged images in the infected brain with DAPI (blue) scale bar = 42 µm. (K) BMNCs were isolated from mice at 4 weeks p.i. and CD11b+ cells were magnetically purified by positive selection. RNA was isolated and reverse transcribed and cDNA was analyzed for levels of Arginase transcript using qRT-PCR. Results are shown as absolute quantitation of Arginase using a standard curve as a ratio to HPRT. Data are represented as mean ± SEM, * p<0.05.

Recent studies have identified a substantial increase in tissue remodeling in the brain during chronic *T. gondii* infection [8]. Additionally, there is a continuous need for the clearance of debris from ruptured cysts and dead cells in the brain [31]. To investigate if AAMØ, known for their role in tissue remodeling and homeostatic clearance, are present during such an event in the CNS, macrophage populations from the infected brain were phenotypically analyzed for the expression of known markers of alternative activation. One of the key molecules that has been associated with a tissue remodeling macrophage phenotype in the CNS is the expression of CXCR3 on microglia [32], [33]. CXCR3 is required for protective immune responses to *T. gondii* primarily due to its role in Th1 cell recruitment and most recently for T cell search strategies in the brain [34]–[37]. Indeed CXCR3 and its ligands are significantly upregulated in the brain at a timepoint associated with significant T cell influx into the CNS following infection (Figure S1A, B) with ∼35% of T cells expressing CXCR3 (Figure 1A). However, in addition to this well characterized role on T cells, there is a small but distinct population of macrophages that express high levels of CXCR3 (∼10% of total macrophages)(Figure 1B). There is also constitutive, although lower, expression of CXCR3 by CNS resident microglia, which remains unchanged following infection (Figure S1C).

To confirm that expression of CXCR3 is associated with alternative activation of macrophages the expression of the scavenger receptor ‘macrophage mannose receptor’ (MMR; also known as Mrc1 and CD206), a key indicator of the AAMØ phenotype [38] was analyzed. Here we show that MMR expression is limited to macrophages and microglia that also express CXCR3 (Figures 1C and S1D). In contrast these cells did not express IL-10 ruling out an anti-inflammatory phenotype (Figure S1E) [39]. Depletion using blocking antibodies to CXCR3 or its ligand, CXCL10 led to a significant decrease in T cell recruitment and a reciprocal increase in parasite burden (Figure S2). However, in addition, the proportion of macrophages in the brain was significantly reduced (Figure 1D) despite no defect in macrophage-attracting chemokines (Figures 1E, F), confirming a role for CXCR3 in the maintenance of this cell population.

To quantify MMR expression by macrophages and microglia in the infected brain, qRT-PCR was performed on magnetically isolated CD11b+ cells from the brains of naive and infected animals. Our results show an approximate 3-fold increase in MMR expression in macrophage populations from infected mice over naïve (Figure 1G). Confirmation of this population in the brain was revealed by immunohistochemical analysis. MMR+ macrophages were observed as small and discrete populations of IBA-1+ or tomato lectin+ cells confirming the source of MMR on macrophages or microglia (Figures 1H, I). A further functional marker of alternative activation is the scavenger receptor stabilin-1 [40]. Stabilin-1 is involved in the clearance of cell corpses as well as the uptake of extracellular matrix components [41], [42]. Expression of MMR co-localized with stabilin-1 and microglia/macrophage markers, confirming that these cells display an alternatively activated phenotype (Figures 1H, I). These cell populations were frequently found in close proximity with intact and degrading *T. gondii* cysts in the CNS (Figure 1J and S3).

An important feature of AAMØ is the cell's ability to produce arginase-1, which acts on its substrate, L-arginine to produce L-ornithine, a precursor to collagen [43]. L-arginine is also the substrate for NO synthase and the two enzymes compete for substrate availability and are regulated by Th1 and Th2 type cytokines [44], [45]. Previous studies have demonstrated that direct infection of macrophages by *T. gondii* tachyzoites can induce arginase expression via STAT-6 dependent and independent pathways [46]–[48]. Furthermore these studies imply that such an induction is a survival strategy enlisted by the parasite to inhibit killing via NO. To assess whether or not macrophages and microglia in the infected brain produce arginase, CD11b+ BMNCs from infected mice were isolated and analyzed for arginase-1 expression by qRT-PCR. Our results show almost a 2-fold increase in arginase-1 expression in cells from infected brains over naïve (Figure 1K). Thus, during chronic *T. gondii* infection there is a population of AAMØ in the CNS characterized by expression of CXCR3, MMR, stabilin-1 and the production of arginase-1.

### Alternatively activated macrophages secrete an active chitinase in the CNS in response to chitin in the cyst wall

**Figure 2:**
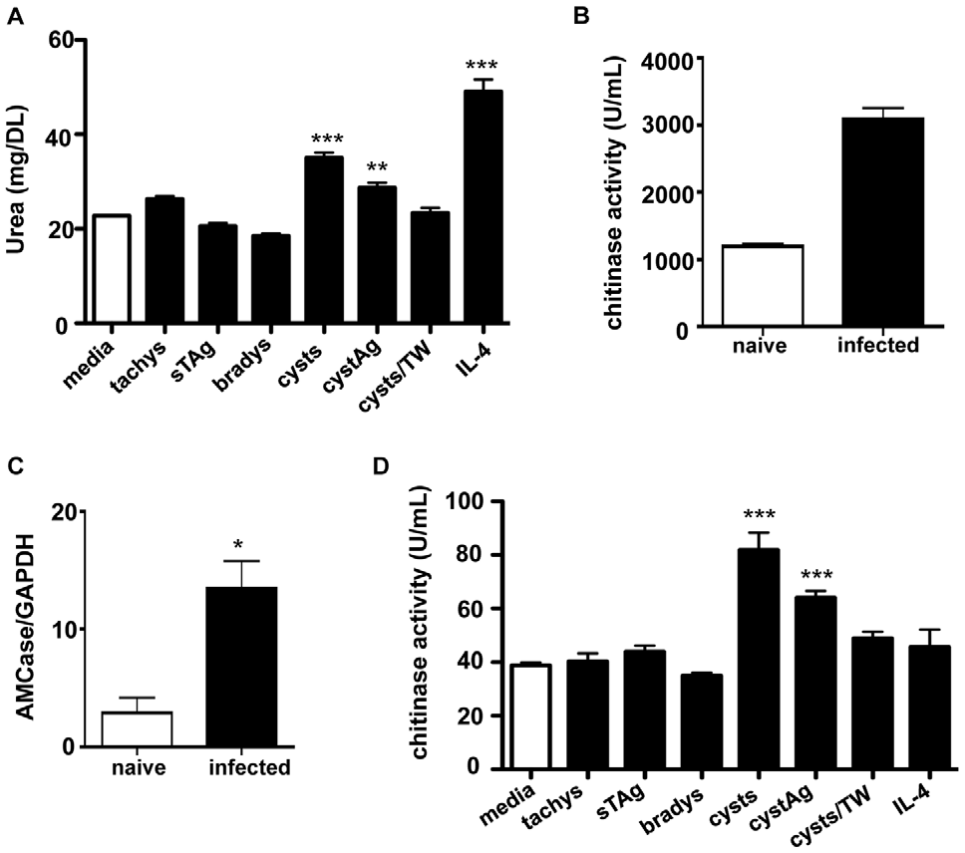
Macrophages produce active chitinase in the CNS in response to the presence of cysts. (A) BMDM were analyzed for arginase activity by measuring urea. BMDM were cultured overnight with tachyzoites, sTAg, bradyzoites, whole cysts, cystAg, cysts separated by 5 µm transwell membranes (cysts/TW), IL-4, or media alone and urea measured colormetrically. (B) C57Bl/6 mice were infected with the Me49 strain of *T. gondii*., sacrificed on day 28 p.i. and lysates from whole brains were analyzed for chitinase activity fluorometrically. (C) qRT-PCR analysis of AMCase expression from whole brain RNA normalized to GAPDH. (D) BMDM were analyzed for chitinase activity fluorometrically. Macrophages were cultured with tachyzoites, sTAg, bradyzoites, whole cysts, cystAg, cysts separated by 5 µm transwell membranes (cysts/TW), IL-4 or media alone. (A–D) Data are representative of at least 3 individual experiments with a minimum of n = 3 and are represented as mean ± SEM, * p<0.05, ** p<0.01, *** p<0.001.

During chronic infection there are several forms of the parasite that could be the source of the infection-associated stimulus for alternative activation of macrophages in the CNS. Since latent cysts are the most prevalent form of infection in the brain, an attractive candidate for the source of this stimulus is the presence of chitin in the cyst wall [49], [50] as it has been shown that the presence of chitin induces the recruitment of macrophages that have an alternatively activated phenotype [51], [52]. To determine if sources of *T. gondii* can induce alternative activation, tachyzoites, bradyzoites, and cysts were added to bone marrow derived macrophage (BMDM) cultures and the production of urea, a downstream product of arginase activity, was measured [53]. In addition, soluble antigen derived from freeze-thawed tachyzoites (sTAg) and whole cysts (cystAg) was assessed for their ability to induce urea (Figure 2A). Our results show a baseline production of urea in unstimulated (media) macrophage cultures, possibly due to the presence of M-CSF [54]. This significantly increased (p<0.001) during AAMØ polarization with IL-4. Despite the known ability of tachyzoites to induce arginase production [46], tachyzoite infection of macrophages did not lead to significant production of urea (Figure 2A). This can be attributed to the use of a type II strain which is a weak inducer of arginase-1 [47], [48]. Importantly, addition of cysts or cyst antigen but not “naked” bradyzoites, did lead to a significant increase in urea production although not as great as induction of alternative activation by IL-4 [38], [55]. This points to components of the cyst wall as the stimulus for AAMØ. Taken together, these data demonstrate that macrophages can be alternatively activated by the presence of *T. gondii* cysts, but not free replicating parasites.

Chitinase activity has been demonstrated in certain populations of AAMØ in both mice and humans [52], [56], [57]. Chitinolytic activity by macrophages has also been implicated in host defense against chitin-containing fungal pathogens [56], [58]. To test whether or not chitinase activity is induced by *Toxoplasma* infection, a chitinase assay was performed on whole brain lysates from naïve and infected animals. Three chitin substrates labeled with 4-methylumbelliferone (4MU) were used to assess the type of chitinase activity present. Upon hydrolysis, 4MU is released and can be measured fluorometrically to determine chitolytic activity. Our data reveal that chitinase activity is significantly increased in the brains of infected mice as compared to the naïve group in only one of the three substrates (Figure 2B). This substrate, 4MU N-acetyl-β-D-glucosaminide, is suitable for detection of exochitinase activity where the enzyme degrades the non-reducing end of the chitin [59]. Several studies have linked chitinases and chitinase-like proteins to inflammation [58]–[60]. This family of 18 glycosyl hydrolases is typically induced during Th2 type immune responses and plays a role in tissue remodeling, fibrosis, and the modulation of both the innate and adaptive immune response [60]. Acidic mammalian chitinase (AMCase) and chitotriosidase (CHIT1) are unique members of this family in that they possess an enzymatically active domain that hydrolyzes the β 1–4 linkages that exist in chitin [56], [58]. Analysis using qRT-PCR demonstrated a significant upregulation of AMCase but not CHIT1. In addition, the chitinase-like protein, Ym-1 (Chi313) was also upregulated following infection (Figure S4). This molecule is known to inhibit IL-12 production and induce alternative activation in macrophages [25], [61], [62]. In contrast to Ym-1, that has been associated with AMCase production by macrophages in the lung and airway [63], Ym-2 and BRP-39 were not upregulated in infected brains (Figure 2C, S4). Since chitin has been shown to activate and recruit AAMØ, it is possible that the cyst wall may serve as the stimulus for chitinase activity in this population of cells. To test this further, BMDM were co-cultured with different forms of the parasite. The addition of tachyzoites, bradyzoites or sTAg did not lead to chitinase production (Figure 2D). In contrast, live cysts and cyst antigen led to a significant increase in chitinase activity that was abolished following chitinase treatment of cysts (Figure 2D, S4). Furthermore, treatment with IL-4 to induce alternative activation in macrophages did not lead to increased chitinase activity. Indeed measurement of IL-4, IL-4Rα and the IL-4-dependent RELM-α [64]–[66] in the brains of chronically infected mice showed no significant increase over naïve mice (Figure S4). These data suggest that the presence of chitin in the cyst wall induces a phenotype of macrophage characterized by the production of the enzymatically active chitinase, AMCase and is distinct from IL-4 induced activation.

Previous work has shown macrophages in close association with rupturing cysts [17], [31] and the presence of an active chitinase could point to a role for these cells in the breakdown of cysts within the brain. Recognition of chitin by macrophages is size dependent and likely contact dependent [49], [58], [67]. To test this, we co-cultured cysts separated from macrophages using 5 µm transwell inserts and assayed for urea and chitinase activity as previously described (Fig. 2A, 2D). Our results show no increase in either urea production or chitinase activity from macrophages that have been separated from cysts, confirming that the observed alternative activation is dependent on contact with cysts or cyst antigen.

**Figure 3:**
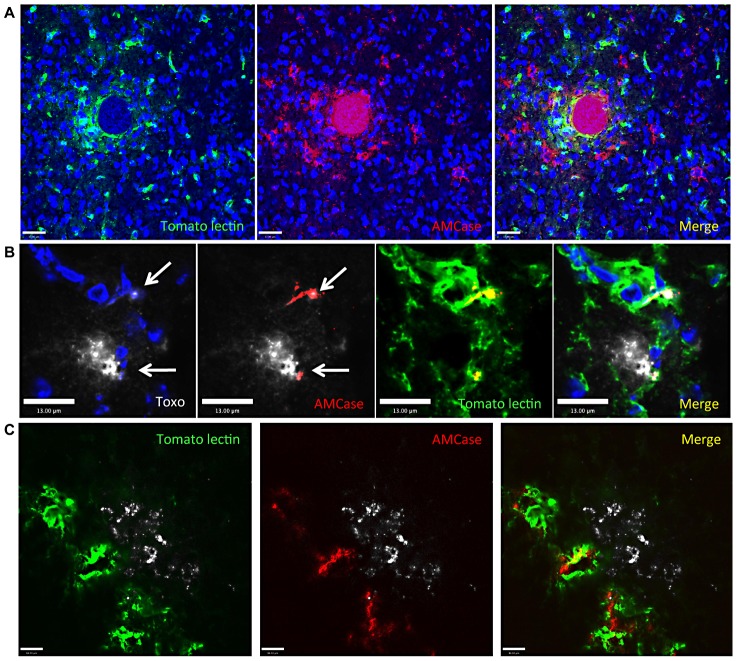
Chitinase expressing AAMØ are in close association with cysts in the CNS. (A–C) Confocal fluorescence microscopy of 20 µm brain slices taken from mice at 4 weeks post infection. Green: tomato lectin labeling macrophages; red: AmCase; blue: DAPI labeling nuclei; white: Toxoplasma. (A) Low magnification (scale bar = 37 µm) (B) High magnification (scale bar = 13 µm) (see also Video S1) arrows point to areas of potential cyst rupture coincident with AMCase expressing macrophages and (C) high magnification (scale bar = 14 µm).

Immunohistochemical analysis of the location of AMCase secreting macrophages in the infected brain shows them in close proximity with tissues cysts (Figures 3A–C). As a proportion of macrophages and microglia in the brain, alternatively activated cells are in the minority and it was not possible to find such cells in the naïve brain. However, cysts are easily identifiable with a highly spherical distinct morphology, can stain non-specifically and specifically with antibodies and individual bradyzoites within the cyst are visible by DAPI staining. Examination of chitinase localization in the infected brain revealed distinct cytoplasmic staining of several cells, nearly all of which were within 75 µm of a cyst (Fig. 3A). Although there were cells that were AMCase positive yet did not stain positively for macrophage/microglial markers, there were clearly several macrophages in close association with cysts that displayed chitinase activity polarized to the cyst wall (Figure S5, arrows). AmCase activity was also observed in macrophages surrounding cysts that seemed to be in the process of lysing or cysts that had been lysed (Figures 3B,C and Video S1). The examples provided show the destruction of the spherical cyst (Video S1) and escaping parasites visualized using anti-Toxoplasma antibodies. Directly at the point of rupture there are AMCase expressing macrophages (Figures 3B). Taken together, these data suggest that the induction of chitinase activity in macrophages occurs in close proximity with the cyst wall and that this distinct population of macrophages is responsible for attacking the long-term chronic cyst stage of Toxoplasma via chitinase activity.

### Macrophage chitinase activity is required to lyse cysts in vitro

**Figure 4:**
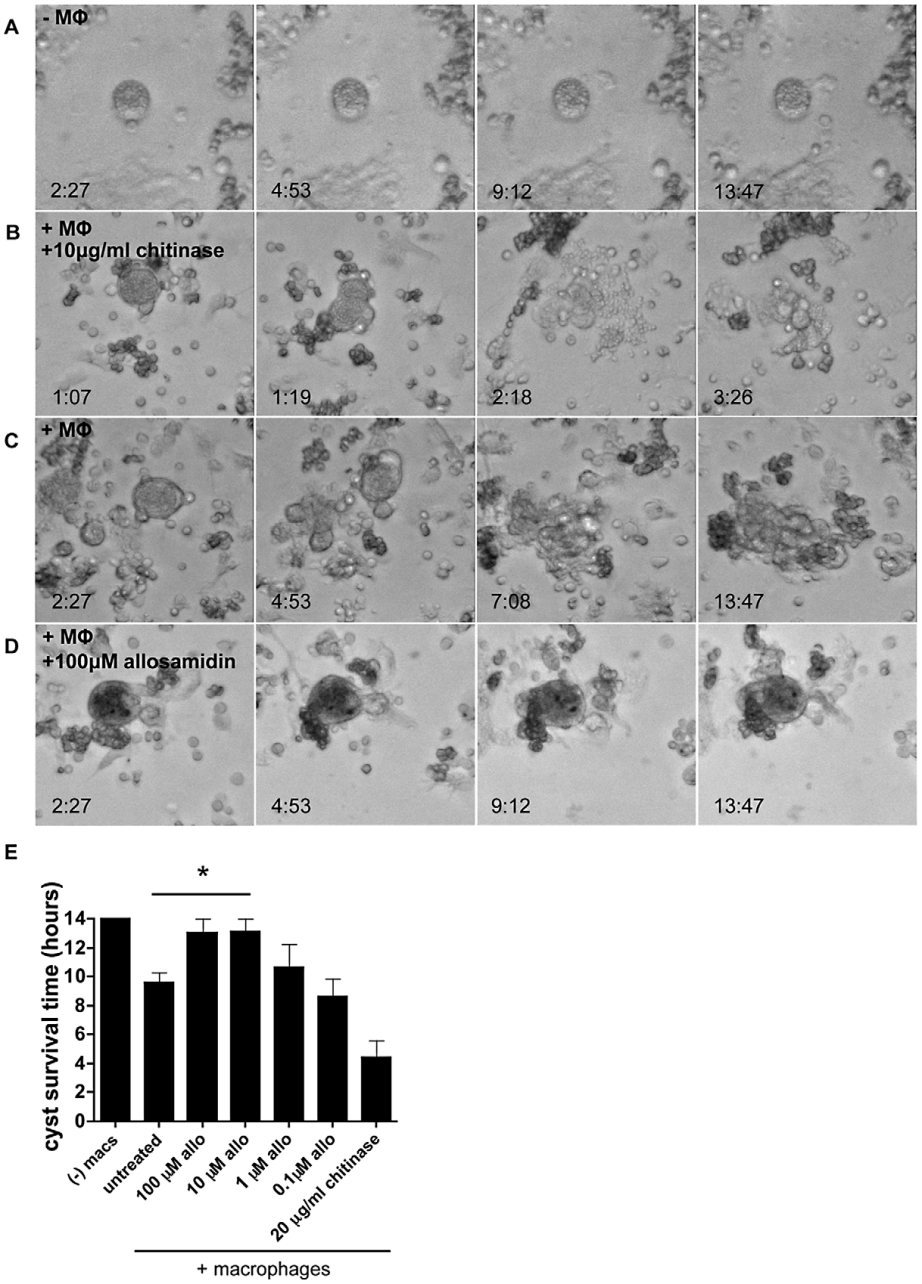
Macrophage chitinase is required to lyse cysts in vitro. (A–E) Cysts purified from the brains of Me49 infected mice were cultured with or without BMDM in a 96 well plate. Cultures were imaged using an HT pathway microscope for 14 hours. Images were collected every 10 minutes and cyst survival time was calculated (A–D) Time-lapse images were taken from (A) cysts alone without macrophages, (B) cysts treated with 10 µg/ml trichoderma chitinase, (C) cysts with macrophages, and (D) cysts with macrophages treated with 100 µM allosamidin. (E) Graph of allosamidin concentration and cyst survival time. Data are representative of at least 3 individual experiments with a minimum of n = 6 and are represented as mean ± SEM, * p<0.05.

The prevailing view is that cysts in the brain remain intracellular within neurons and that CD8+ T cell production of perforin is responsible for cyst clearance in the brain although the exact mechanism of cyst destruction has yet to be described [17], [18]. In order to determine whether or not macrophage chitinase activity could be responsible for the direct lysis of cysts, BMDM were co-cultured with cysts; with and without the chitinase inhibitor allosamidin. Cultures were observed for 14 hours capturing images every 10 minutes. Cysts observed in the absence of macrophages remained intact for the entire time course suggesting no parasite intrinsic mechanism of cyst destruction (Figure 4A; Video S2). In contrast, the addition of 20 µg/ml trichoderma chitinase to cyst cultures led to rapid rupture of the cysts within an average of 4 hrs, releasing bradyzoites into the media (Figure 4B; Video S3). Strikingly, cysts that were cultured with macrophages came under vigorous attack. This involved efficient and rapid migration of macrophages toward the cyst creating clusters of macrophages that could be seen pulling at the cyst wall (Figure 4C; Video S4). In these cultures most of the cysts were destroyed during the observation period with the average survival time of 9.5 hours (Figure 4E). In contrast cysts cultured in the presence of macrophages and the chitinase inhibitor allosamidin survived significantly longer than in untreated cultures. Although there appeared to be no defect in the recruitment and activity of macrophages to cysts with similar clustering and ‘pulling’ of the cyst wall, the majority of cysts survived the entire 14 hour period when treated with either 100 µM or 10 µM allosamidin (Figure 4D; Video S5). Decreasing concentrations of allosamidin led to a dose dependent decrease in cyst survival time (Figure 4E). These results demonstrate that macrophages can induce cyst lysis in a chitinase dependent manner.

**Figure 5:**
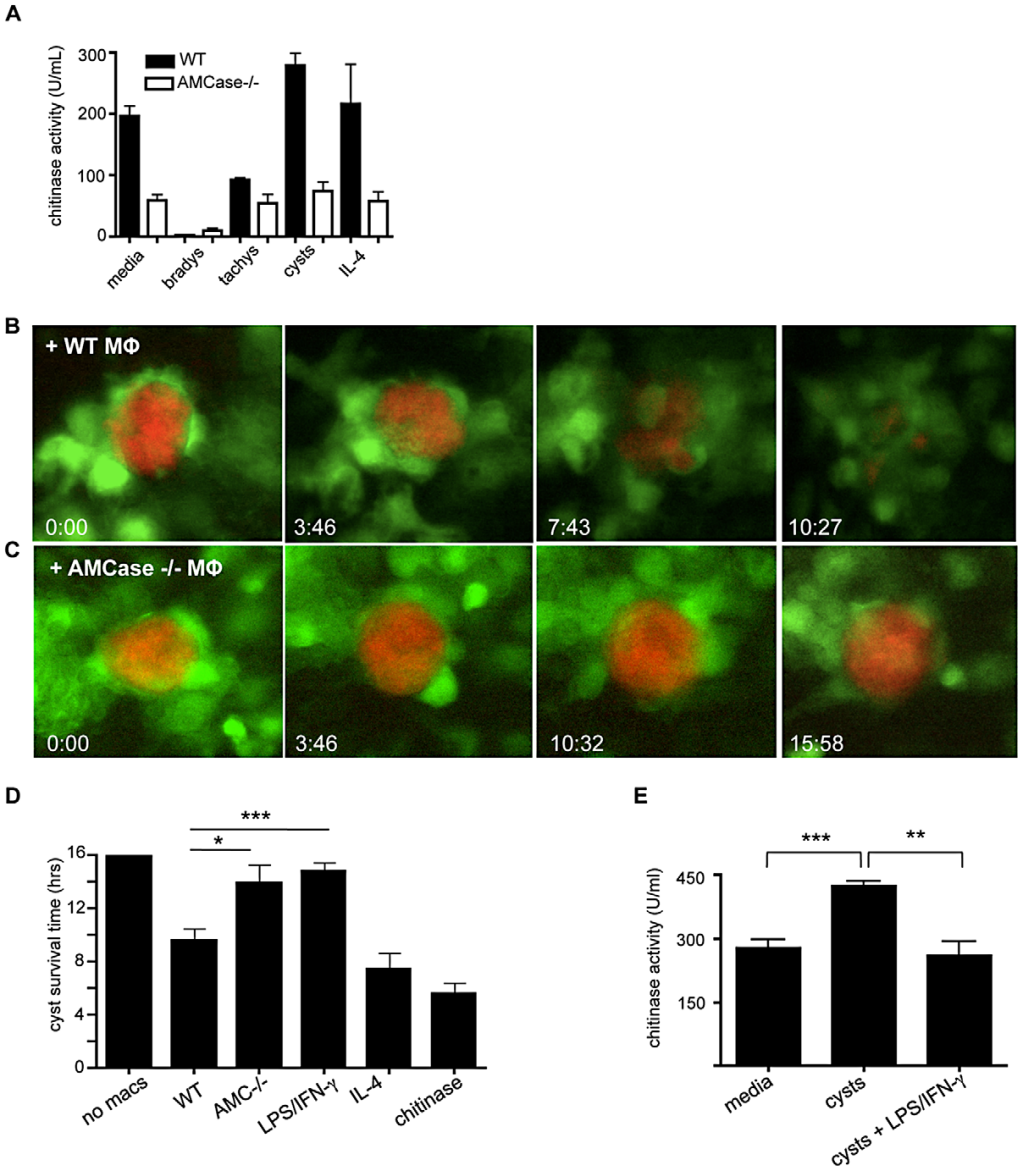
Acidic mammalian chitinase is required to lyse cysts in vitro. (A) BMDM from WT and AMCase−/− mice were analyzed fluorometrically for chitinase activity. Chitinase activity from WT and AMCase−/− macrophages cultured with bradyzoites, tachyzoites, whole cysts, IL-4 or media alone. (B–D) Me49-RFP cysts purified from the brains of infected mice were cultured with or without CellTracker green labeled BMDM from either WT or AMCase null mice. Cultures were imaged using an HT pathway microscope for 16 hours. Images were collected every 10 minutes and cyst survival time was calculated. (B–C) Time-lapse images were taken from cyst cultures (B) WT macrophages or (C) AMCase−/− macrophages. (D) Graphical representation of cyst survival time over 16 hours. (E) Chitinase activity from WT bone marrow-derived macrophages cultured with whole cysts, whole cysts+LPS/IFN-γ, or media alone. Data are representative of at least 3 individual experiments with a minimum of n = 6 and are represented as mean ± SEM, * p<0.05, ** p<0.01 *** p<0.001.

Although both AMCase and CHIT1 are upregulated in certain bacterial and nematode infections [68], [69] only AMCase was significantly increased in the brain following Toxoplasma infection (Figure 2C). To confirm that AMCase is responsible for the observed chitinase activity, we performed a chitinase assay similar to that in Figure 1D using BMDM from WT and AMCase null mice (Figure 5A). Our results reveal a severe defect in chitinase production by AMCase null macrophages. Indeed, these cells showed a significantly lower baseline level of chitinase and were unresponsive to the addition of cysts. To test whether the ability to destroy cysts is dependent on this enzyme, BMDM from WT and AMCase−/− mice were fluorescently labeled and cultured with Me49-RFP expressing cysts and cyst lysis time imaged as before. Using fluorescently labeled parasites enhanced the ability to see escaping parasites from lysing cysts. Results, as before, demonstrated that WT non-polarized macrophages were able to lyse cysts in ∼10 hours (Figures 5B and 5D; Videos S6 and S10). In contrast, cysts cultured with AMCase−/− macrophages had a significantly increased survival time over WT macrophages consistent with AMCase being the source of chitinase activity required to lyse cysts (Figures 5C and 5D; Videos S7 and S11). To determine the requirement for macrophage polarization in their ability to lyse cysts, macrophages were treated with cytokines to polarize them to classical or alternative phenotypes prior to cyst addition. In line with the lack of chitinase induction, IL-4 priming had no effect on cyst survival time, suggesting that cytokine-induced alternative activation does not enhance the ability to destroy cysts (Figures 5D; Video S9). In contrast, macrophages that were classically polarized with LPS and IFN-γ showed a defect in chitinase activity and cyst destruction (Figures 5D and 5E; Video S9). Suggesting that polarization of macrophages is required but that chitin is the most likely source of alternative activation and not IL-4. Taken together, these data demonstrate that macrophages lyse cysts in an AMCase-dependent manner *in vitro*.

### AMCase dependent control of parasite burden in vivo

**Figure 6:**
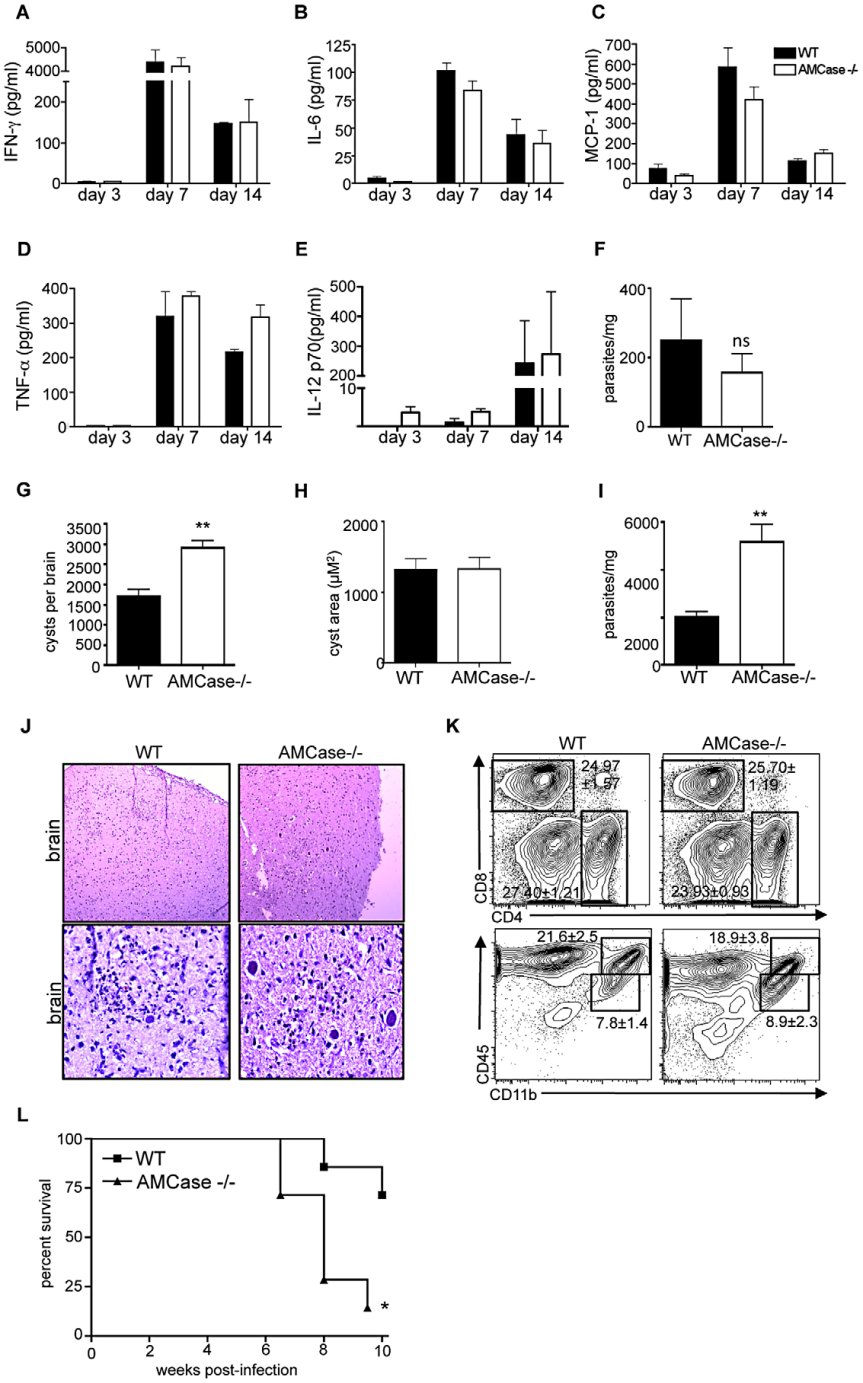
AMCase−/− mice have a higher parasite burden in the brain and succumb to infection during the chronic stage. (A–F) C57Bl/6 (WT) and AMCase−/− mice were infected with the Me49 strain of *T. gondii*. Serum was isolated from whole blood samples at days 3, 7 and 14 post infection and analyzed for (A) IFN-γ, (B) IL-6, (C) MCP-1, (D) TNF-α, (E) IL-12p70 (F) At day 7 DNA was isolated from the lungs and analyzed for parasite burden using qPCR. Results are displayed as parasites per mg tissue. (G–K) C57Bl/6 (WT) and AMCase−/− mice were infected with the Me49 strain of *T. gondii* and sacrificed at 5 weeks following infection. Brains were harvested and analyzed for cyst burden, cellular composition and histology. (G) Cyst counts were obtained from homogenized whole brain samples. (H) Cyst area, 20 cysts from each mouse were photographed microscopically and cyst area was determined using ImageJ software. (I) DNA from brains of WT and AMCase−/− was isolated and analyzed for parasite burden using qPCR. (J) Whole brains were fixed, frozen and stained for H&E to examine cyst burden and pathology. (K) BMNCs were isolated and analyzed for expression of CD4+ T cells, CD8+ T cells, macrophages (CD45^hi^/CD11b+) and microglia (CD45^hi^/CD11b+) by flow cytometry. Significance was determined using log rank test with p = 0.0177. Data are representative of at least 2 individual experiments with a minimum of n = 4 and are represented as mean ± SEM, ns = not significant, * p<0.05, ** p<0.01. (L) Survival data from C57Bl/6 (squares, n = 7) and AMCase−/− (triangles, n = 7). Data are representative of 4 individual experiments with C57Bl/6 (n>40) and AMCase−/− (n = 40) and significance tested using Log-rank (Mantel-Cox) and Gehan-Breslow Wilcoxan test * p<0.05.

The consequences of chitinase dependent cyst lysis in the CNS could potentially benefit either the host or the parasite. If the escaping bradyzoites were quickly killed by macrophages or associated immune cells, we would expect this mechanism to benefit the host and result in a lower parasite burden. Conversely, if bradyzoites are able to propagate and infect new cells, this could be a mechanism that promotes the persistence of the parasite in the brain. To investigate the role of AMCase in the brain *in vivo*, we infected WT and AMCase deficient mice and analyzed the immune response and parasite burden in the absence of chitinase activity. To determine if AMCase is required during the acute stage of infection, tissue samples from lungs were taken at day 7 and analyzed for parasite burden by qPCR. No significant differences in lung parasite load were found and serum cytokine concentrations were equivalent throughout acute infection (Figure 6A–F). Thus a lack of AMCase does not lead to deficient immune responses early on during infection in the periphery. At 5 weeks post infection, when systemic inflammation has been controlled and parasites are located solely in the brain predominantly as cysts containing bradyzoites [67], [70] parasite burden was evaluated. In the absence of AMCase, there was a significant increase (p = 0.0014) of approximately 2-fold in the total number but not the size of cysts in the brain (Figures 6G and 6H). Differences in cyst burden were not observed at 3 weeks post infection (Figure S6), a period representing the transition between acute and chronic infection, further suggesting that the increase in cyst burden is occurring due to events within the CNS during chronic infection. In addition, total parasite burden in the CNS as measured by qPCR was significantly greater by more than 2-fold (p = 0.0055) (Figure 6I) correlating with the appearance of more cysts histologically (Figure 6J). In addition, parasite burden was evaluated using RT-qPCR with stage-specific primers identifying tachyzoite (SAG1), bradyzoite (SAG4), and cyst (MAG1) specific transcripts [71](Figure S6). Our results show similar increases in parasite burden for all three transcripts, suggesting that cyst lysis is also an important mechanism to control the cell invasive forms of the parasite. Flow cytometric analysis revealed no differences in infiltrating CD4+, CD8+ T cells, or macrophage populations (Figure 6K). Therefore, the increase in parasite burden is not due to a defect in infiltrating effector immune cells. Furthermore, AMCase−/− mice failed to survive and succumbed to infection beginning at six weeks (p = 0.0177) (Figure 6L). Although some acute mortality was noted over several experiments significance was only achieved when chronic mortality was included. These results demonstrate that AMCase activity is required for the protective immune response to *T. gondii* during chronic infection in the brain and that AMCase mediated cyst lysis in the CNS is a beneficial mechanism for the host to control parasite burden at non-lethal levels.

## Discussion

Chronic infections represent a continuous battle between the host's immune system and pathogen replication. Many protozoan parasites and fungal pathogens have evolved a cyst lifecycle stage that provides it with increased protection from environmental degradation as well as endogenous host mechanisms of attack [72]–[74]. In the case of Toxoplasma, these cysts are predominantly found in the immune protected brain making clearance of the parasite more difficult and resulting in a lifelong infection. Here we describe three novel findings 1) despite a dominant Th1 immune response during Toxoplasma infection there exists a population of macrophages in the infected brain which display a distinct alternatively activated phenotype; 2) these cells are responsible for chitinase dependent lysis of Toxoplasma cysts and 3) this chitinase activity is through the production of AMCase which is required for protective immune responses.

Multiple studies have demonstrated the role of CXCR3 and its ligands in the migration of activated T cells during Th1 immune responses including to sites of infection [75], [76]. It is also known that the chemokines CXCL9 and CXCL10 are induced by the presence of the proinflammatory cytokine, IFN-γ [34], [36], [37], [77]. More recently, however, the function of this family of chemokines has expanded to include neural-glial signaling following brain lesion where injured neurons upregulate CXCL10 and recruit CXCR3 expressing microglia to phagocytose denervated dendrites [33]. Consistent with this, another recent study has implicated CXCR3 in the function of perivascular macrophages and their ability to remodel the vasculature during stress [78]. We noted upregulation of CXCR3, CXCL9 and CXCL10 in the brain during chronic Toxoplasma infection. Furthermore, CXCR3 was preferentially expressed on macrophages expressing the scavenger receptors MMR and stabilin-1, suggesting an alternatively activated phenotype for these cells. Previous studies have established important functions of AAMØ in the context of helminth infection and wound healing [43], [79], [80] however not during an infection that generates such a polarized Th1 immune response such as Toxoplasma. *T. gondii* is known to exploit the arginine metabolic pathway and induce arginase-1 expression in macrophages thereby suppressing nitric oxide production but this does not lead to the alternative activation of these cells [46]–[48]. Instead our data point to a role for the cyst being the source of alternative activation of macrophages and the subsequent ability of these cells to lyse cysts via destruction of the chitin in the cyst wall. Thus, we observed a contact dependent significant increase in arginase activity following treatment with cysts and cystAg suggesting that this induction is not a result of infection by the replicating parasite, but rather by the presence of chitin in the cyst wall. The weak induction of arginase activity observed also points to a limited role for arginase-1 in the chitin-induced phenotype.

Chitin is found in the exoskeletons of insects, fungal cell walls, sheaths of parasitic nematodes, and is a component of the *T. gondii* cyst wall [49], [50]. The presence of this exogenous molecule can induce the recruitment of AAMØ, basophils, neutrophils, and eosinophils [52], [81]–[84]. Active chitinases such as AMCase and chitotriosidase are secreted by macrophages in response to chitin-containing pathogens and has been shown to inhibit hyphal growth of chitinous fungi such as *Candida* and *Aspergillus* [56], [58]. Despite the link between chitin and the recruitment of type 2 inflammation in the lung [52], a recent study has demonstrated no role for AMCase in the generation of allergic airway pathology [85]. In this study we demonstrate for the first time, macrophage chitinase activity in response to a protozoan pathogen. Chitin recognition is thought to be a size dependent process and involve a combination of TLR2 and scavenger receptors such as MMR and dectin-1 [49], [58]. Here we have demonstrated the presence of such scavenger receptors in association with cysts and it will be of interest in future studies to determine the role of these molecules in cyst containment during Toxoplasma infection. Independent of the receptors involved it is likely that this is a contact-dependent process and indeed, cysts were unable to induce urea production or chitinase activity in macrophages when separated by transwell membranes. Furthermore, analysis of the location of AMCase producing cells in the brain finds them reliably close to cysts and often in direct contact with reactivating or rupturing cysts. These images show that despite the presence of many DAPI positive cells surrounding the cyst structure, the escape of parasites through the cyst wall occurs juxtaposed to the macrophage or AMCase activity. Our data suggest that the presence of cyst antigens induces alternative activation of macrophages and that these antigens are required for macrophages to produce chitinase even in the presence of IL-4. Thus alternative activation of macrophages is not sufficient for AMCase production and chitinase activity. The significant increase of the non-enzymatically active chitinase-like molecule YM-1 in infected brains is consistent with previous reports of AMCase and YM-1 being co-expressed specifically in macrophages and not epithelial cells [63].

Live imaging *in vitro* demonstrated AMCase dependent degradation of cysts as shown using both a chitinase inhibitor and AMCase−/− macrophages. Although there is no evidence of an active chitinase produced by *T. gondii* (ToxoDB), the similar cyst survival times observed for AMCase-null macrophages and chitinase-inhibited macrophages exclude the possibility that bradyzoites are the source of enzymatic activity and are breaking out of the cyst. The chitin dependent induction of chitinase activity implies that macrophages have access to the chitin in the cyst wall prior to chitinase-mediated cyst destruction. The prevailing view from ultrastructure studies is that cysts remain intracellular within neurons [15], [86], [87](*A. Koshy and J. Boothroyd personal communication*) yet analysis of cyst burden over time shows a reduction in cyst numbers implying some form of effector mechanism in place [16]. Several studies have demonstrated perforin dependent control of cyst burden during chronic infection [17], [18]. We suggest that instead of a direct effect of perforin on cysts, it is most likely that perforin production by CD8+ T cells may initiate this process by lysing the cyst infected cell, thus exposing the cyst wall to chitinase activity from macrophages. This model would explain the many observations of macrophages in close association with rupturing cysts [8], [17], [19]. Of note, we found that BALB/c macrophages that are more easily alternatively activated had enhanced cyst lysis activity when compared to C57Bl/6 (Figure S7). This may be one explanation for the increased resistance to toxoplasmic encephalitis in BALB/c mice [30], [88].

Although AMCase activity is not required for protective immunity during acute infection it is required for protection during the chronic stage of infection. Our observation of a higher cyst count and parasite burden as well as decreased survival in AMCase-null mice points to a specific and important role for chitinase mediated cyst lysis in the brain. Thus, within the brain, cyst containment seems as important as the killing of free parasites in the control of pathology. In addition, continuous chitin-mediated attack by macrophages and the release of parasites from latent cysts will provide a constant source of antigenic stimulation for the immune response. This latter discovery may provide an explanation for the continuous recruitment of T cells into the brain.

It has been apparent for some time that cyst numbers in the brain can be controlled endogenously yet identification of the exact effector mechanisms has not been so apparent. In these studies we demonstrate the presence of a distinct population of macrophages in the brain during chronic Toxoplasma infection, which express CXCR3, MMR, stabilin-1 and arginase-1. Furthermore these macrophages have chitinase activity, are localized to cysts and are observed in association with cyst degradation. The mechanism of cyst lysis is dependent on AMCase and this enzyme is required for survival during chronic infection to reduce parasite burden. The continuous presence of Toxoplasmic cysts in the brain and other tissues presents a constant threat of reactivation to the immune compromised patient. Mechanisms that enhance cyst removal or prevent their reactivation during Toxoplasma or other protozoan infections would provide a novel line of anti-parasitic therapies.

## Methods

### Ethics statement

The experiments in this study were performed in strict accordance with the recommendations in the Guide for the Care and Use of Laboratory Animals of the National Institutes of Health. The protocols were approved by the Institutional Animal Care and Use Committee at University of California, Riverside. All efforts were made to minimize animal suffering during the course of these studies.

### Mice and parasites

*T. gondii* Pruigniund and RH strains were maintained *in vitro* as previously described [89]. Soluble toxoplasma antigen (sTAg) was prepared from RH strain tachyzoites as previously described [90]. The Pruigniud strain was used for *in vitro* tachyzoite infections at a ratio of 3∶1. The Me49 strain of *T. gondii* was maintained in infected Swiss Webster and CBA/CaJ mice. For infection, brains from infected mice were removed placed in 3 ml sterile 1×PBS and passed 3–5 times through an 18.5 gauge followed by 20.5 and 22.5 gauge needle. The number of cysts in a 30 µl aliquot was determined microscopically. Brain suspensions were adjusted to 100 cysts/ml and mice were infected each with 20 cysts intraperitoneally. Infection studies of C57Bl/6 and AMCase null mice were conducted at least four times with a minimum of 7 biological controls. C57Bl/6, CBA/CaJ (Jackson, Bar Harbor, ME) and Swiss Webster mice (Charles River, Wilmington, MA) were maintained in a pathogen free environment under IACUC established protocols at the University of California Riverside. AMCase-null mice were generated by targeting exon 5 using loxP/CRE recombination as previously described [85]. The AMCase gene deleted mice were of a mixed background, C57BL/6NTac:129SvEvBrd, and were backcrossed to C57/BL6 for at least 10 generations. These mice were generated and maintained under IACUC protocols established by Pfizer.

### Preparation of splenocyte and brain mononuclear cell (BMNCs) suspensions

A single cell suspension from spleens was prepared by passing through a nylon 40 µm cell strainer (BD, San Jose, CA). Suspensions were washed with RPMI complete (10% FCS, 1%Pennicilin/Streptomycin, 1% Glutamine, 1% Sodium Pyruvate, 1% nonessential amino acids, 0.1% B-mercaptoethanol) (Life Technologies, Grand Island, NY) and centrifuged for 5 minutes at 1200 RPM at 4°C. Red Blood Cells were lysed using 0.86% ammonium chloride solution, centrifuged and resuspended in RPMI complete. BMNCs were prepared as previously described [89].

### Flow cytometry

BMNCs or splenic cells were incubated with various conjugated antibodies against CXCR3, CD3, CD4, CD8, CD11b, IL-10, and CD45, (eBioscience, San Diego, CA) and MMR (Biolegend, San Diego, CA). Cells were analyzed using the BD FACSCanto II flowcytometer (BD Biosciences, San Jose, CA) and FlowJo analysis software v.8.7.3 (Treestar Software, Ashland, OR). Cell populations were determined by gating on CD4+, CD8+, CD45^hi^/CD11b+ (macrophages) and CD45^int^/CD11b+ (microglia) from live cell gate.

### Quantitative Reverse transcription PCR (qRT-PCR)

Total RNA from brain tissue samples was extracted with TRIzol reagent (Life Technologies, Grand Island, NY) according to manufacturer's instructions. DNase1 treatment and first strand cDNA synthesis was performed using cDNA synthesis kit (BioRad, Hercules, CA) according to the manufacturer's instructions. CXCR3, CXCL9, CXCL10, CCL2, CCL5, AMCase, Arg1, and Chit1 specific primers for Real Time PCR were purchased from IDT's primer Quest (http://www.idtdna.com/Scitools/Applications/Primerquest/). Primer sequences were as follows: CXCR3, forward, 5′-TGTAGCCCTCACCTGCATAGTTGT-3′; reverse, 5′-GTTGTACTGGCAATGGGTGGCATT-3′, CXCL9, forward, 5′- TCAGATCTGGGCAAGTGTCCCTTT-3′; reverse, 5′-TTTGGTGACGTGAGCCTCAGAAGT-3′, CXCL10, forward, 5′- TGGCTAGTCCTAATTGCCCTTGGT-3′; reverse, 5′- TCAGGACCATGGCTTGACCATCAT-3′, CCL2 forward, 5′-TCACCTGCTGCTACTCATTCA-3′; reverse, 5′-TACAGCTTCTTTGGGACACCT-3′, CCL5 forward, 5′-TCGTGCCCACGTCAAGGAGTA-3′; reverse, 5′-TCTTCTCTGGGTTGGCACACA-3′, AMCase forward, 5′-TTTCCACTTCTCAGAACCGCC-3′; reverse, 5′-TGTTGCTCTCAATAGCCTCCT-3′, CHIT1 forward, 5′-AGTTCGGTTCTTTCCCAGGGA-3′; reverse, 5′- GCTGATGGTTGTCCATTCCAG -3′, Arg1 forward, 5′-TGGCTTTAACCTTGGCTTGCT-3′; reverse, 5′- AAAGAACAAGCCCTTGGGAGG-3′, SAG1 forward, 5′- CGACAGCCGCGGTCATTCTC-3′; reverse, 5′- GCAACCAGTCAGCGTCGTCC-3′, SAG4 forward, 5′-CTGCTTTCGTCTGTCTTCAAC-3′; reverse, 5′-CTTCTTCACTGGCAATGAACTC-3′, MAG1 forward, 5′-TGAGAACTCAGAGGACGTTGC-3′; reverse, TCTGACTCAAGCTCGTCTGCT-3′ Ym1 forward, 5′-CCTGCCTGTGTACTCACCTG-3′; reverse, 5′-AGCCTTGGAATGTCTTTCTCCA-3′; Ym2 forward, 5′-CCTGCCTGTGTACTCACCTG-3′; reverse, 5′-CAGCCTTGGAATGTGGTTCA-3′; RELM-α forward, 5′-GTCAGCAATCCCATGGCGTA-3′; reverse, 5′-GGCCCATCTGTTCATAGTCTTG-3′; BRP-39 forward, 5′-TTACCAGACGCCATCCAACC-3′; reverse, 5′-ATAAGAACGCAGGAACGGGG-3′; IL-4Rα forward, 5′-AGCCAGGACTGGGACTAGAG-3′; reverse, 5′-CCTCGAGGTATCGCCTTGAC-3′; IL-4 forward, 5′-CCATATCCACGGATGCGACA-3′; reverse, 5′-AAGCCCGAAAGAGTCTCTGC-3′. Real-time PCR was performed using the iQ5 real-time PCR Detection System (Bio-Rad, Hercules, CA) in total 25 µl reaction mixture with 12.5 µl SYBR Green qPCR Master Mix (2×) (Bio-Rad, Hercules, CA) and 300 nM primer. The reaction conditions were as follows: 10 min at 95°C, followed by 40 cycles of 15 s at 95°C and 60 s at 60°C. The HPRT (*Hypoxine* Phosphoribosyl- Transferase) forward primer (5′-CCCTCTGGTAGATTGTCGCTTA-3′) and reverse primer (5′- AGATGCTGTTACTGATAGGAAATTGA -3′) were used as an endogenous control. Quantified results represent the fold induction of target gene expression at different days post infection in comparison to the target gene expression in naïve cDNA samples. Analysis on 1.5% agarose gels was performed to exclude nonspecific amplification. NTC, no-template control (reagent alone without template) was included in each assay to detect any possible contamination of the PCR reagents.

### Immunohistochemistry

Immediately following excision, brains were bisected sagitally and flash-frozen in cold isopentane. Frozen brains were then put into standard Tissue-Tek cryomold and filled with Optimal Cutting Temperature (OCT) solution (Tissue-Tek, Torrance, CA) and put on dry ice and subsequently stored at −80°C. Serial sections of 10–20 µm were prepared on a standard Cryostat machine (LEICA/CM1850, Simi Valley, CA). Frozen tissue sections were fixed 75% acetone/25% ethanol then blocked in 10% donkey serum prior to incubation with purified antibodies. Purified primary antibodies for Iba-1 (Wako, Richmond, VA), CXCR3 (Life Technologies, Grand Island, NY), MMR (AbD Serotec, Raleigh, NC) arginase-1, AMCase and stabilin-1 (Santa Cruz Biotechnology, Santa Cruz, CA) as well as biotinylated tomato lectin (Sigma-Aldrich, St. Louis, MO) were incubated with tissue samples for 2 h at RT or overnight at 4°C, and followed with appropriate secondary antibodies conjugated to Alexa 488, Alexa 568, or Alexa 647 at 2 µg/mL (Life Technologies, Grand Island, NY). Samples were mounted in Prolong Gold with DAPI (Life Technologies, Grand Island, NY) for nuclear counterstaining. Images were collected on a Leica SP2 scanning confocol microscope (Leica Optics, Germany), and analyzed using Improvision Volocity 5.0 (Perkin-Elmer, Waltham, MA). Distance of cells from cysts were calculated from confocal images of at least 12 cysts and at least 6 AMCase expressing cells per cyst.

### Quantification of *T. gondii* burden by quantitative PCR (qPCR)

Parasite burden was measured by amplifying the *T. gondii* genes B1, SAG1, SAG4, or MAG1 by real-time PCR as previously described [71], [89].

### In vivo peptide blocking

C57BL/6 mice were infected i.p. with 10^4^ Pruigniund tachyzoites. At day 21, 23, 25, and 27 post infection the animals were injected i.p. with either 0.5 ml α-CXCL10 (0.5 mg/mL), 0.5 mL α-CXCR3 (polyclonal), or 0.5 ml PBS as previously published [76], [91]. The mice were sacrificed on day 28 p.i. and brains were excised for flow cytometric analysis, and parasite burden as described above. Blocking studies were conducted twice with at least 5 biological replicates.

### Urea assay

Supernatants from infected macrophage cultures were added to a 96 well UV plate at 50 µl per sample in triplicates. Urea reagents A and B were mixed from quantichrom urea assay kit (Bioassay systems, Hayward, CA) and 200 µl of mixture added to each well. Included standard was used starting at 50 mg/ml and diluted 2 fold. Samples were incubated for 30 min at room temperature and plates were read at 520 nm to determine urea concentration.

### Chitinase assay

In order to quantitate chitinolytic activity, 10 µg of protein from lysates was added to the fluourogenic substrates 4-Methylumbelliferyl N,N′-diacetyl-β-D chitobioside, 4-Methylumbelliferyl N-acetyl-β-D-glucosaminide, or 4-Methylumbelliferyl β-D-N,N′,N″-triacetylchitotriose (Sigma-Aldrich, St. Louis, MO). The samples were incubated at 37°C for 30 minutes and the reactions were stopped by adding 200 µl sodium carbonate. The fluorescent intensity of free 4-methylumbelliferone (4MU) was measured on a fluorimeter at excitation of 360 nm and emission of 450 nm. A standard curve was generated using serial dilutions of 4MU.

### Bone marrow derived macrophages

Femurs and tibias were obtained from 6–12 week old C57BL/6 mice. After euthanasia, the mice were sprayed with 70% ethanol and the femurs and tibias were dissected using scissors. Muscles connected to the bone were removed using scissors, and the femurs were placed into a 50 mL tube containing sterile DMEM on ice. In a tissue culture hood, the bones were washed in sterile DMEM and then both epiphyses were removed using sterile scissors and forceps. The bone marrow was flushed out with a 10 ml syringe filled with BM20 differentiation media (DMEM supplemented with 10% fetal bovine serum, 20% L929 supernatant, 5% horse serum, 100 U/ml penicillin, 100 µg/ml streptomycin, and 2 mM L-glutamine) (Life Technologies, Grand Island, NY) into a 50 mL sterile tube. The tube was vortexed gently and topped off to 50 mL with fresh BM20. 10 mL of cell suspension was plated out on 100 cm untreated dishes and incubated for 7 days at 37°C, 5% CO_2_ with fresh media added at day 4. Cells were then washed, counted and plated at 10^6^ cells/mL in BM10 media (DMEM supplemented with 10% fetal bovine serum 10% L929 supernatant, 5% horse serum, 100 U/ml penicillin, 100 µg/ml streptomycin, and 2 mM L-glutamine) (Life Technologies, Grand Island, NY) into a 50 mL sterile tube and allowed to rest for 3 days. Macrophages were stimulated overnight with either recombinant IL-4 (10 ng/ml), LPS (50 ng/ml) or IFN-γ (100 U/ml) (all from R&D Systems, Minneapolis, MN), stAg (100 µg/ml) or cystAg (100 µg/ml) in complete DMEM.

### Macrophage/cyst assays

To observe the interaction of macrophages and cysts *in vitro*, cysts were isolated from the brains of chronically infected mice as described above. 50 cysts were added per well to 96 well plates containing 2×10^5^ bone marrow derived macrophages. Cysts and cells were viewed using a BD HT Pathway 855 microscope (BD Biosciences, San Jose, CA) in a climate-controlled chamber (37°C, 5% CO_2_). Nine cysts were identified per condition and photographed every 10 minutes for 14 or 16 hours. Movies were compiled using ImageJ software (NIH, Bethesda, MD) and cyst survival time was determined.

### Statistics

For statistical analysis of survival data, the log-rank and Gehan-Breslow Wilcoxon test was used and involved over 40 C57Bl/6 and 40 AMCase−/− mice. Acute (0–14 days) and chronic deaths (>14days) were analyzed individually. For all other data, an unpaired, two-tailed Student's t test, or ANOVA test with a 95% confidence interval was used (Prism; GraphPad Software, Inc., La Jolla, CA). All data are represented as means ± SEM.

## Supporting Information

Figure S1 **CXCR3 expression on the surface of AAMØ.** C57Bl/6 (WT) mice were infected with the Me49 strain of *T. gondii* and sacrificed at various timepoints following infection. (A–B) RNA was isolated from infected brains, reverse transcribed, and the resulting cDNA was analyzed for CXCR3, CXCL9 and CXCL10 transcript levels using qRT-PCR. Results are shown as (A) absolute quantitation using standard curve as a ratio to HPRT, and (B) relative quantitation (ΔΔCt) shown as fold increase over naïve. (C–E) BMNCs were isolated from the brains of naïve and 4 week infected mice and analyzed by flow cytometry. (C) Microglial (CD45^int^/CD11b+) expression of CXCR3 from naïve and infected mice. (D) Microglial (CD45^int^/CD11b+) expression of MMR on CXCR3+ and CXCR3− populations. (E) Intracellular staining for IL-10 expression by macrophages (CD45^hi^/CD11b+) expressing CXCR3 with isotype control (left panel). Data are representative of at least 2 individual experiments with a minimum of n = 3 and are represented as mean ± SEM.
(TIF)

Figure S2 **CXCR3 is a functional receptor on the surface of macrophages in the infected CNS.** (A–C) C57Bl/6 mice were infected with the Me49 strain of *T. gondii*., neutralizing antibodies for CXCR3 and CXCL10 were administered beginning at 21 days post infection and mice were sacrificed on day 28 after infection. (A) BMNCs were isolated from treated and untreated mice and total cell counts were obtained using hemocytometer. (B) BMNCs were stained and analyzed for cellular composition using flow cytometry. The proportion of CD3+ cells was multiplied by total BMNC count for absolute quantitation. (C) DNA was isolated from the brains of treated and untreated mice and parasite burden was determined by qPCR analysis. Data are representative of at least 2 individual experiments with a minimum of n = 3 and are represented as mean ± SEM, * p<0.05, ** p<0.01.
(TIF)

Figure S3 **AAMØ associated with cyst lysis.** Confocal fluorescence microscopy of 20 µm brain slices taken from mice at 4 weeks post infection. Imunohistochemical analysis of alternatively activated macrophage (Iba-1, red) as judged by its expression of stabilin-1 (green), adhering closely to a large round cyst. Polarized, to the site of macrophage ‘attachment’, bradyzoites are seen escaping in an organized fashion towards or into the AAMØ (arrows).
(TIF)

Figure S4 **Chitinase activity is dependent on the presence of chitin and is independent of IL-4 activation.** A) Bone marrow derived macrophages were analyzed for chitinase activity. Macrophages were cultured with whole cysts, cysts treated with trichoderma chitinase or media alone. Data are representative of at least 2 individual experiments with a minimum of n = 3 and are represented as mean ± SEM. B) qRT-PCR was conducted on BMNC to measure YM-1, YM-2, RELM-a, BRP39, IL-4 and IL4Ra. Data are presented as fold increase over naïve.
(TIF)

Figure S5 **AMCase activity associated with cyst.** Confocal fluorescence microscopy of 20 µm brain slices taken from mice at 4 weeks post infection. Immunohistochemical analysis of macrophage (Iba-1, green) and AMCase (red), arrows point to AMCase polarized to the cyst wall.
(TIF)

Figure S6 **AMCase−/− polarization and infection studies.** BMDM from WT and AMCase−/− mice were polarized to A) M2 and B) M1 phenotype as measured by Urea and Greiss assays respectively. C57Bl/6 (WT) and AMCase−/− mice were infected with the Me49 strain of *T. gondii* and sacrificed at C) 3 weeks for cyst counts and D) 5 weeks following infection. RNA was isolated from infected brains, reverse transcribed, and the resulting cDNA was analyzed for SAG1, SAG4, and MAG1 transcript levels using qRT-PCR to measure gene expression from tachyzoites, bradyzoites and cysts, respectively. Results are shown as absolute quantitation of copy number using standard curve. Data are representative of at least 3 individual experiments with a minimum of n = 3 and are represented as mean ± SEM, ** p<0.01, *** p<0.001.
(TIF)

Figure S7 **BALB/c macrophages lyse cysts more quickly than C57Bl/6 macrophages.** BMDM from BALB/c and C57Bl/6 mice were cultured with cysts and imaged using an HT pathway microscope for 16 hours. Images were collected every 10 minutes and cyst survival time was calculated.
(TIF)

Video S1 **3D colocalization of AMCase-secreting macrophages and parasite cysts.** Three dimensional z-plane progression of 20 µm confocal image taken from mice at 4 weeks post infection. Green: tomato lectin labeling macrophages; red: AmCase; blue: DAPI labeling nuclei; white: anti-Toxoplasma.
(MOV)

Video S2 **Cysts do not lyse in the absence of macrophages.** 14 hour time-lapse movie of cysts cultured in the absence of BMDM. Images were collected every 10 minutes and movies were compiled using ImageJ.
(MOV)

Video S3 **Rupture of** ***T. gondii*** **cysts in the presence of chitinase.** 14 hour time-lapse movie of cysts co-cultured with WT BMDM and pretreated with 10 µg/ml trichoderma chitinase. Images were collected every 10 minutes and movies were compiled using ImageJ.
(MOV)

Video S4 **Untreated Cysts cultured with bone marrow-derived macrophages.** 14 hour time-lapse movie of cysts co-cultured with untreated BMDM. Images were collected every 10 minutes and movies were compiled using ImageJ.
(MOV)

Video S5 **Cysts cultured with BMDM and treated with allosamidin.** 14 hour time-lapse movie of cysts co-cultured with WT BMDM and treated with 100 µM allosamidin. Images were collected every 10 minutes and movies were compiled using ImageJ.
(MOV)

Video S6 **Cysts cultured with untreated WT BMDM.** 16 hour time-lapse movie of cysts co-cultured with BMDM from WT mice. Images were collected every 10 minutes and movies were compiled using ImageJ.
(MOV)

Video S7 **Cysts cultured with untreated AMCase-null BMDM.** 16 hour time-lapse movie of cysts co-cultured with BMDM from AMCase-null mice. Images were collected every 10 minutes and movies were compiled using ImageJ.
(MOV)

Video S8 **Cysts cultured with WT BMDM pretreated with LPS/IFN-γ.** 16 hour time-lapse movie of cysts co-cultured with BMDM from WT mice, pre-treated with LPS/IFN-γ. Images were collected every 10 minutes and movies were compiled using ImageJ.
(MOV)

Video S9 **Cysts cultured with WT BMDM pretreated with IL-4.** 16 hour time-lapse movie of cysts co-cultured with BMDM from WT mice, pre-treated with IL-4. Images were collected every 10 minutes and movies were compiled using ImageJ.
(MOV)

Video S10 **Me49-RFP cysts cultured with WT BMDM labeled with CellTracker green.** 16 hour time-lapse movie of Me49-RFP cysts co-cultured with CellTracker green labeled BMDM from WT mice. Images were collected every 10 minutes and movies were compiled using ImageJ.
(MOV)

Video S11 **Me49-RFP cysts cultured with AMCase-null BMDM labeled with CellTracker green.** 16 hour time-lapse movie of Me49-RFP cysts co-cultured with CellTracker green labeled BMDM from AMCase-null mice. Images were collected every 10 minutes and movies were compiled using ImageJ.
(MOV)
