# Supplementary figures and images for: Chitinase Dependent Control of Protozoan Cyst Burden in the Brain
Source: PLoS Pathog. 2012 Nov 29;8(11):e1002990. doi: 10.1371/journal.ppat.1002990 (PMC3510238; doi:10.1371/journal.ppat.1002990)

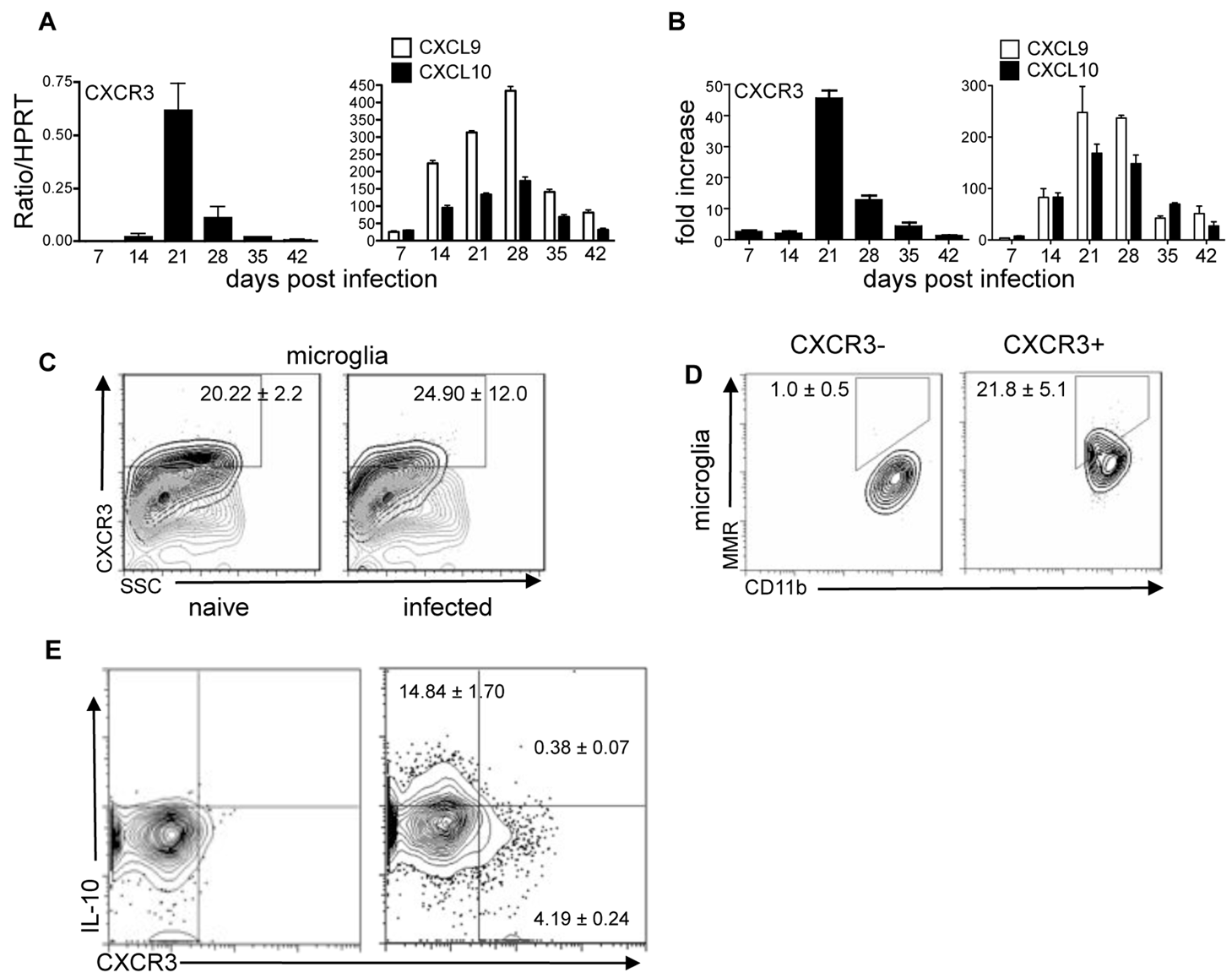

Supplement: Figure S1 — CXCR3 expression on the surface of AAMØ. C57Bl/6 (WT) mice were infected with the Me49 strain of T. gondii and sacrificed at various timepoints following infection. (A–B) RNA was isolated from infected brains, reverse transcribed, and the resulting cDNA was analyzed for CXCR3, CXCL9 and CXCL10 transcript levels using qRT-PCR. Results are shown as (A) absolute quantitation using standard curve as a ratio to HPRT, and (B) relative quantitation (ΔΔCt) shown as fold increase over naïve. (C–E) BMNCs were isolated from the brains of naïve and 4 week infected mice and analyzed by flow cytometry. (C) Microglial (CD45int/CD11b+) expression of CXCR3 from naïve and infected mice. (D) Microglial (CD45int/CD11b+) expression of MMR on CXCR3+ and CXCR3− populations. (E) Intracellular staining for IL-10 expression by macrophages (CD45hi/CD11b+) expressing CXCR3 with isotype control (left panel). Data are representative of at least 2 individual experiments with a minimum of n = 3 and are represented as mean ± SEM. (TIF) [file ppat.1002990.s001.tif]

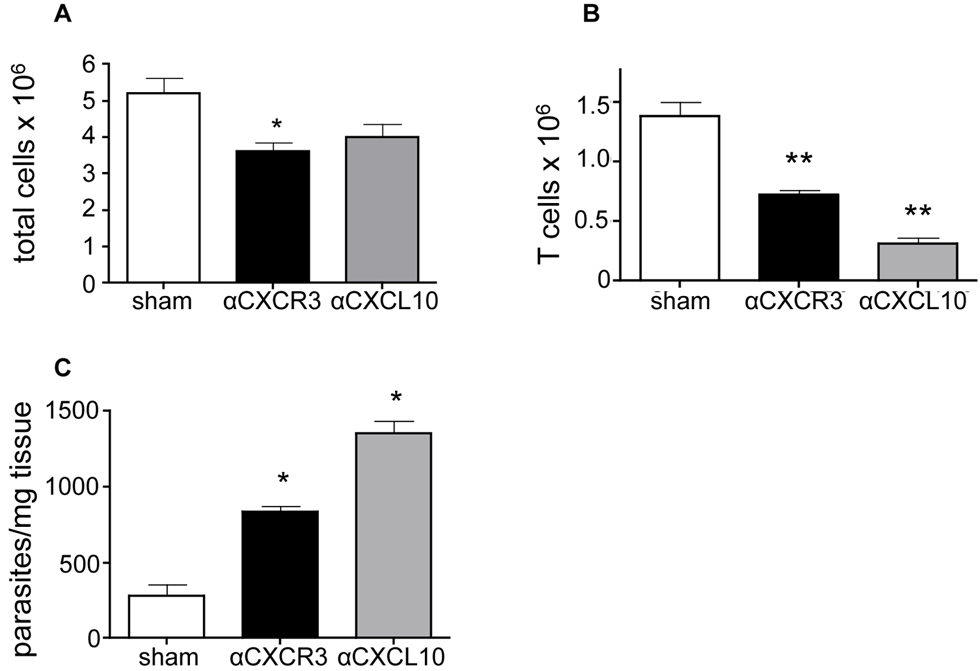

Supplement: Figure S2 — CXCR3 is a functional receptor on the surface of macrophages in the infected CNS. (A–C) C57Bl/6 mice were infected with the Me49 strain of T. gondii., neutralizing antibodies for CXCR3 and CXCL10 were administered beginning at 21 days post infection and mice were sacrificed on day 28 after infection. (A) BMNCs were isolated from treated and untreated mice and total cell counts were obtained using hemocytometer. (B) BMNCs were stained and analyzed for cellular composition using flow cytometry. The proportion of CD3+ cells was multiplied by total BMNC count for absolute quantitation. (C) DNA was isolated from the brains of treated and untreated mice and parasite burden was determined by qPCR analysis. Data are representative of at least 2 individual experiments with a minimum of n = 3 and are represented as mean ± SEM, * p<0.05, ** p<0.01. (TIF) [file ppat.1002990.s002.tif]

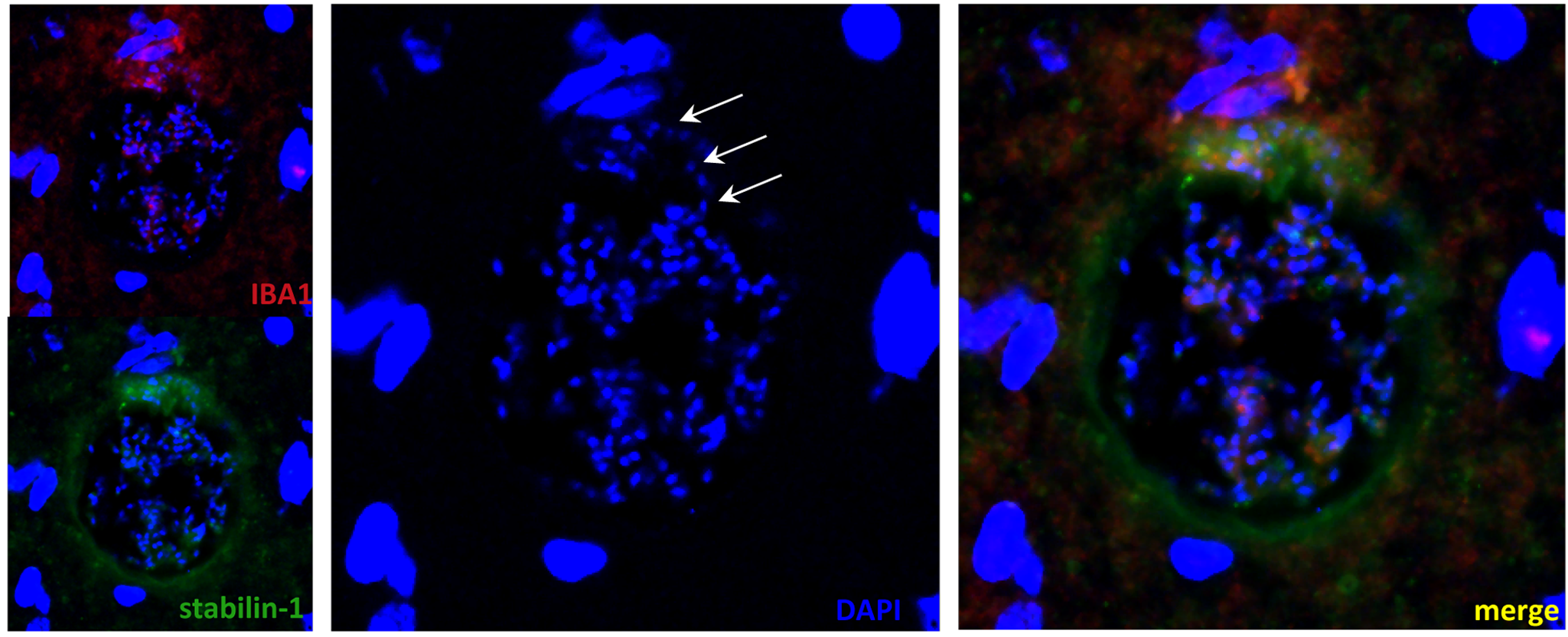

Supplement: Figure S3 — AAMØ associated with cyst lysis. Confocal fluorescence microscopy of 20 µm brain slices taken from mice at 4 weeks post infection. Imunohistochemical analysis of alternatively activated macrophage (Iba-1, red) as judged by its expression of stabilin-1 (green), adhering closely to a large round cyst. Polarized, to the site of macrophage ‘attachment’, bradyzoites are seen escaping in an organized fashion towards or into the AAMØ (arrows). (TIF) [file ppat.1002990.s003.tif]

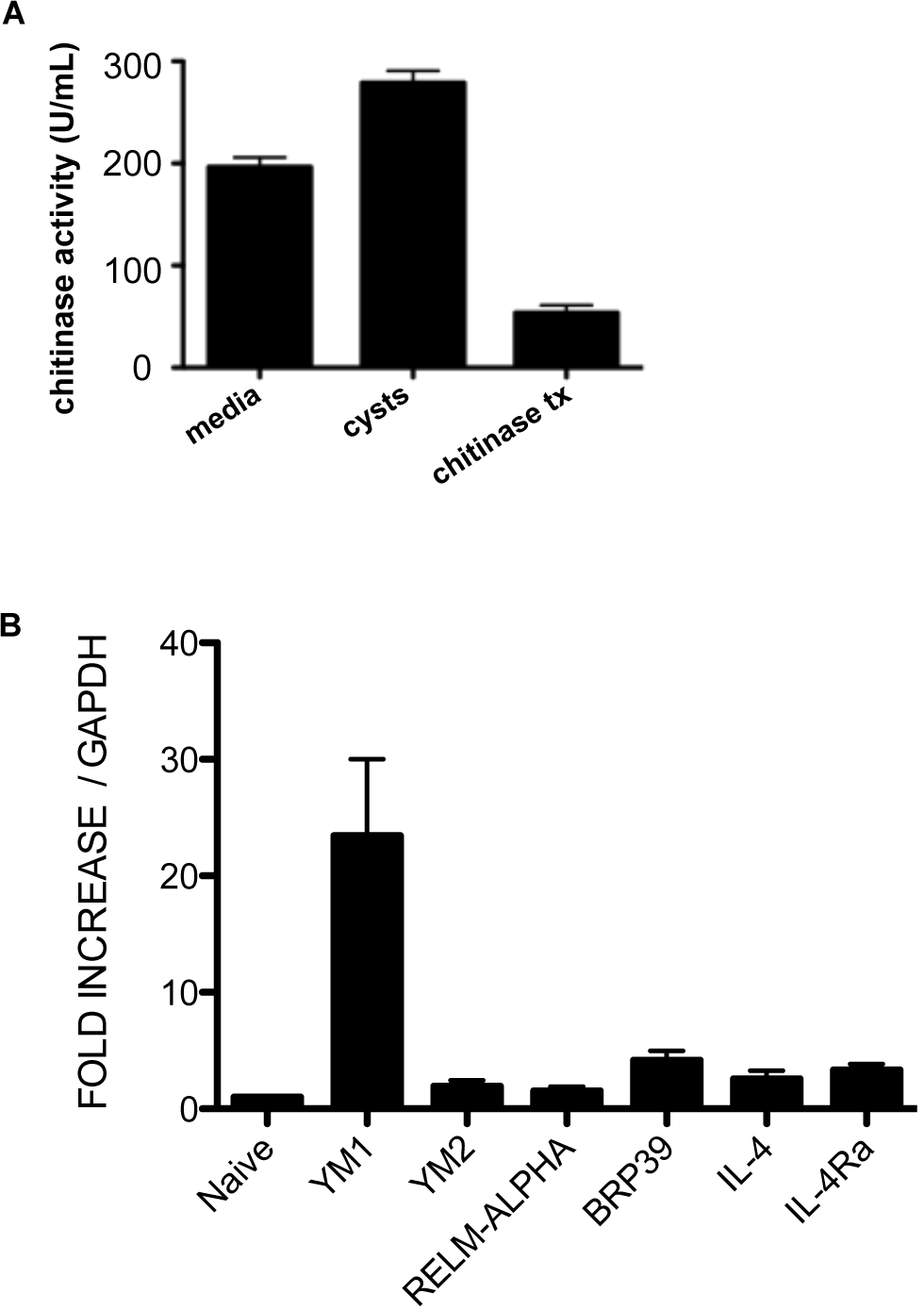

Supplement: Figure S4 — Chitinase activity is dependent on the presence of chitin and is independent of IL-4 activation. A) Bone marrow derived macrophages were analyzed for chitinase activity. Macrophages were cultured with whole cysts, cysts treated with trichoderma chitinase or media alone. Data are representative of at least 2 individual experiments with a minimum of n = 3 and are represented as mean ± SEM. B) qRT-PCR was conducted on BMNC to measure YM-1, YM-2, RELM-a, BRP39, IL-4 and IL4Ra. Data are presented as fold increase over naïve. (TIF) [file ppat.1002990.s004.tif]

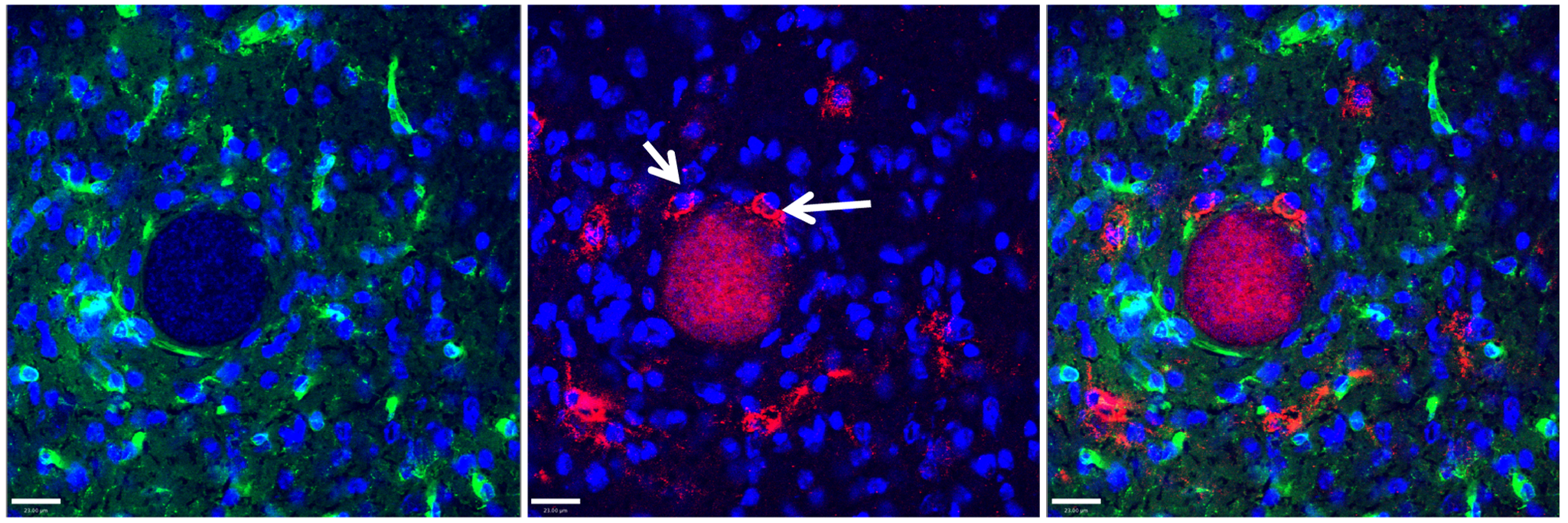

Supplement: Figure S5 — AMCase activity associated with cyst. Confocal fluorescence microscopy of 20 µm brain slices taken from mice at 4 weeks post infection. Immunohistochemical analysis of macrophage (Iba-1, green) and AMCase (red), arrows point to AMCase polarized to the cyst wall. (TIF) [file ppat.1002990.s005.tif]

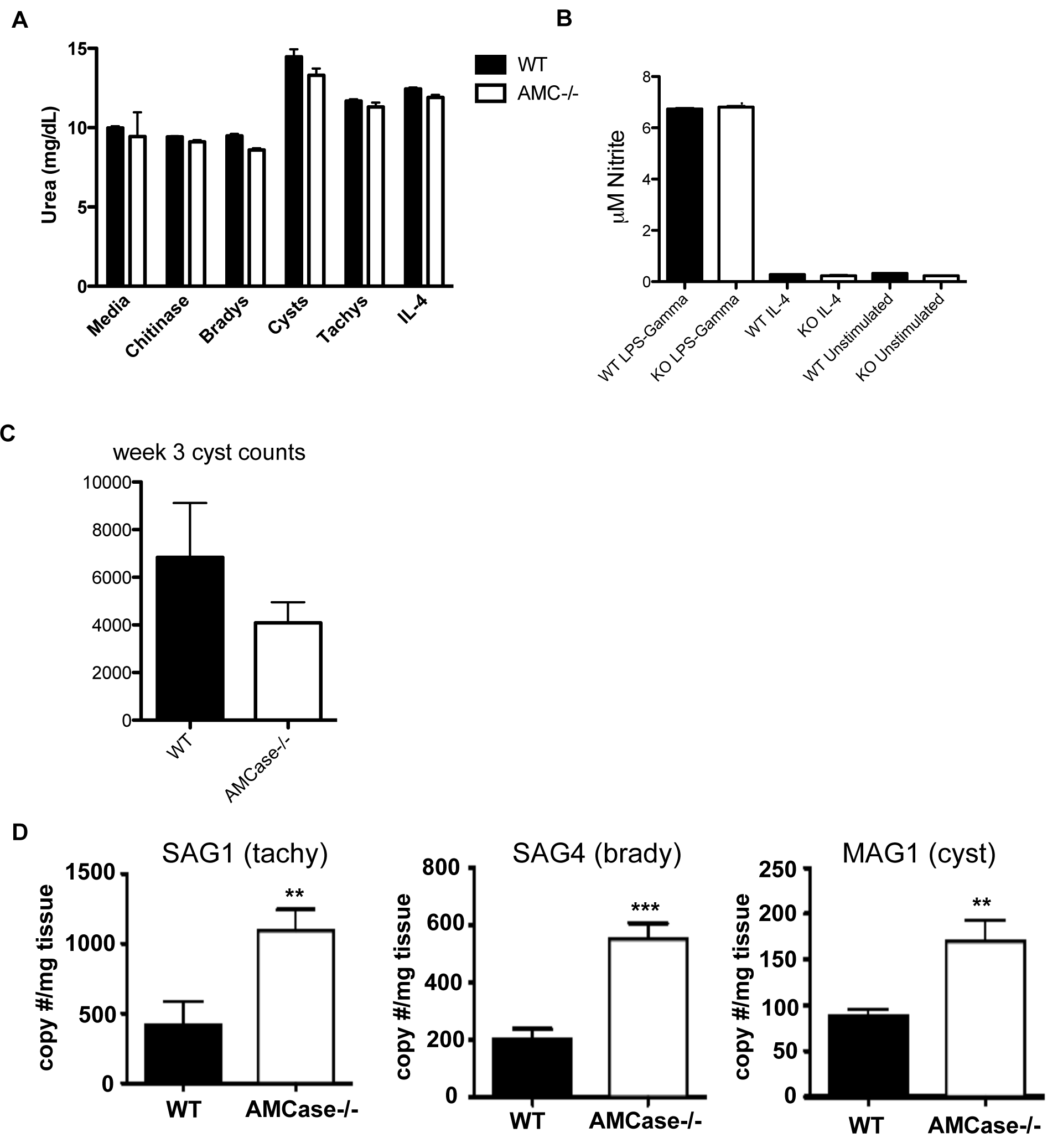

Supplement: Figure S6 — AMCase−/− polarization and infection studies. BMDM from WT and AMCase−/− mice were polarized to A) M2 and B) M1 phenotype as measured by Urea and Greiss assays respectively. C57Bl/6 (WT) and AMCase−/− mice were infected with the Me49 strain of T. gondii and sacrificed at C) 3 weeks for cyst counts and D) 5 weeks following infection. RNA was isolated from infected brains, reverse transcribed, and the resulting cDNA was analyzed for SAG1, SAG4, and MAG1 transcript levels using qRT-PCR to measure gene expression from tachyzoites, bradyzoites and cysts, respectively. Results are shown as absolute quantitation of copy number using standard curve. Data are representative of at least 3 individual experiments with a minimum of n = 3 and are represented as mean ± SEM, ** p<0.01, *** p<0.001. (TIF) [file ppat.1002990.s006.tif]

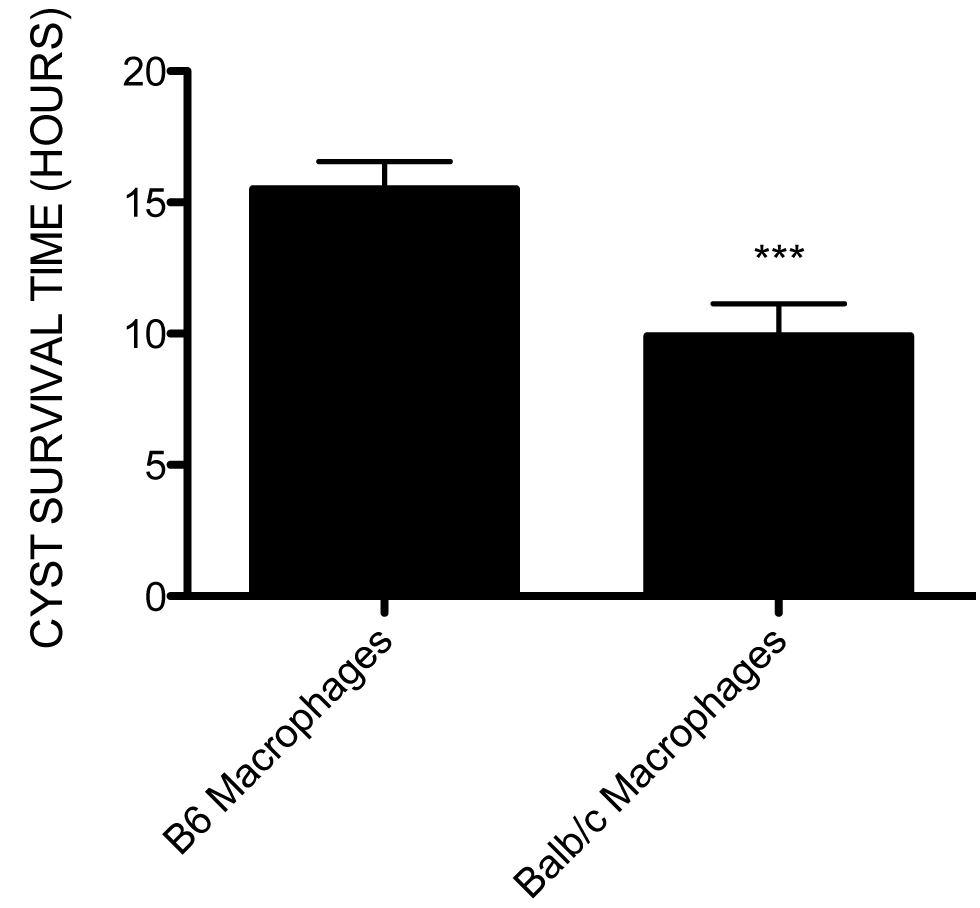

Supplement: Figure S7 — BALB/c macrophages lyse cysts more quickly than C57Bl/6 macrophages. BMDM from BALB/c and C57Bl/6 mice were cultured with cysts and imaged using an HT pathway microscope for 16 hours. Images were collected every 10 minutes and cyst survival time was calculated. (TIF) [file ppat.1002990.s007.tif]
